# Uncovering the genetic structure and evolutionary history of *Acanthopagrus latus* (Actinopterygii, Sparidae) using mitochondrial DNA and multivariate analyses: A case study of coastal populations in Central Vietnam

**DOI:** 10.3897/BDJ.13.e164274

**Published:** 2025-08-22

**Authors:** Ty Nguyen, Van Loi Bui, Le Thuy Lan Hoang, Thi Kim Anh Tran, Xuan Huy Nguyen, Van Giang Tran

**Affiliations:** 1 Faculty of Biology, University of Education, Hue University, Hue, Vietnam Faculty of Biology, University of Education, Hue University Hue Vietnam; 2 Hue University, Hue, Vietnam Hue University Hue Vietnam; 3 School of Agriculture and Natural Resources, Vinh University, Vinh City, Vietnam School of Agriculture and Natural Resources, Vinh University Vinh City Vietnam; 4 Sydney Institute of Agriculture & School of Life and Environmental Sciences, The University of Sydney, NSW, Australia Sydney Institute of Agriculture & School of Life and Environmental Sciences, The University of Sydney NSW Australia; 5 Department of Science, Technology and International Relations, Hue University, Hue, Vietnam Department of Science, Technology and International Relations, Hue University Hue Vietnam

**Keywords:** Central Vietnam, control region, population structure, yellowfin seabream

## Abstract

The yellowfin seabream (*Acanthopagrus latus*), a commercially important marine fish in Vietnam, faces threats from overfishing and habitat degradation and requires a deeper understanding of its genetic structure and evolutionary history for effective conservation. This study investigated the genetic diversity, population structure and demographic history of *A. latus* across three populations in Central Vietnam using mitochondrial control region (mtCR) sequences and multivariate analyses. A total of 125 fish samples were collected between January 2023 and June 2024, with DNA extracted from pectoral fin tissues and amplified for mtCR sequencing. Phylogenetic and haplotype network analyses revealed three main haplogroups with high genetic diversity (haplotype diversity: 0.993 ± 0.002), but minimal population differentiation (Fst = 0.00029, p = 0.39188), indicating significant gene flow, facilitated by coastal currents as these populations maintain strong genetic connectivity with the coastal populations of southern China. Neutrality tests, mismatch distribution and Bayesian Skyline Plots have suggested a historical population expansion followed by recent stability and slight decline. These findings highlight the species’ resilience, but underscore the need for regionally coordinated conservation strategies to protect critical habitats like estuaries and mangroves, ensuring the sustainability of *A. latus* populations in Vietnam.

## Introduction

In the face of accelerating environmental change, overexploitation of marine resources and habitat degradation, the study of population genetics has emerged as a crucial tool for understanding species resilience, evolutionary potential and for providing information for effective conservation strategies. At the core of population genetics lies the quantification of genetic variation within and amongst populations, providing insights into historical demography, gene flow and the adaptive capacity of species under both natural and anthropogenic pressures (Islam et al. 2022, Sun et al. 2022). In particular, in marine systems, where many species exhibit high dispersal capabilities and complex life cycles, genetic data are essential for identifying population structure that is not always evident from morphology or geography alone.

The yellowfin seabream (*Acanthopagrus latus*), a member of the family Sparidae, is a protandrous hermaphrodite fish widely distributed across the Indo-West Pacific Region, from Japan and China to the coasts of Southeast Asia and northern Australia. It is of high commercial value in both capture fisheries and aquaculture and plays a significant ecological role in estuarine and nearshore environments (Wang et al. 2020, Tang et al. 2023). In Vietnam, *A. latus* is a vital component of artisanal fisheries, particularly in coastal provinces of the Central Region. However, increasing fishing pressure, habitat contraction due to coastal development and pollution pose threats to its long-term sustainability (Yan et al. 2021, Peng et al. 2023). Despite its economic importance, knowledge of the population genetic structure and evolutionary history of *A. latus* in Vietnam remains limited.

Mitochondrial DNA (mtDNA) and, specifically, the control region (mtCR), has been widely used to assess intraspecific genetic variation in marine organisms due to its high mutation rate, lack of recombination and maternal inheritance. This marker allows for the detection of historical demographic events, such as population expansions, contractions or bottlenecks (Duong et al. 2023). In the case of *A. latus*, previous studies have revealed cryptic genetic lineages and population structuring across its distribution range, suggesting that, despite high dispersal potential, population connectivity may be limited by environmental factors, ocean currents or habitat discontinuities (Islam et al. 2022, Li et al. 2025). In addition, recent advancements in phylogeographic analysis and Bayesian modelling now enable the reconstruction of species’ demographic histories in unprecedented detail. Tools, such as Bayesian Skyline Plots (BSP) and haplotype network analyses, help to visualise past population dynamics and identify potential refugia or expansion fronts, providing crucial context for conservation planning (Bouckaert et al. 2019, Cheng et al. 2019). Moreover, fixation indices (Fst) and AMOVA provide statistical quantification of genetic differentiation across spatial scales, offering insights into the extent of gene flow and the potential existence of evolutionarily significant units. The application of population genetics to conservation biology is particularly vital for marine fishes, where traditional stock assessment methods may fail to detect underlying genetic structure or demographic history. Genetic data can provide information for the design of marine protected areas, guide restocking programmes and help assess the risks of inbreeding or genetic erosion in exploited populations (Liu et al. 2021). In this context, understanding the genetic structure of *A. latus* populations in Central Vietnam not only contributes to basic evolutionary knowledge, but also has direct implications for sustainable fisheries management, stock enhancement programmes and habitat conservation efforts.

The Vietnamese coastline represents a dynamic and ecologically diverse region influenced by monsoonal cycles, freshwater inputs and complex current systems, such as the Kuroshio and North Equatorial Currents. These oceanographic factors likely play a pivotal role in shaping the genetic structure of coastal marine species through larval dispersal and connectivity (Nguyen et al. 2022). Central Vietnam’s monsoonal cycles, with distinct wet (September–December) and dry (January–August) seasons, induce pronounced seasonal variations in precipitation, river discharge and coastal hydrology. These variations result in fluctuating salinity, temperature and nutrient levels, which profoundly influence the distribution, reproduction and survival of coastal marine species like *A. latus* (Nguyen et al. 2013, Do et al. 2018, Tran et al. 2022). Furthermore, estuarine and coastal environments, characterised by significant salinity fluctuations during the rainy season, function as essential nursery grounds for aquatic organisms including *A. latus* (Truong et al. 2010, Do 2022). Interconnected with seagrass beds and mangrove forests, these estuaries enhance regional biodiversity, providing vital habitats that support the life cycles of numerous aquatic species (Nguyen et al. 2022).

Yet, localised retention mechanisms, such as estuarine nursery dependency or habitat specificity, may act in opposition to homogenising forces, resulting in genetic differentiation at surprisingly fine geographic scales (Wang et al. 2024). *A. latus* exhibits a strong preference for estuarine environments for juvenile development, where low salinity, abundant food resources and sheltered conditions provide optimal nursery grounds (Sourinejad et al. 2015, Tran et al. 2019). This estuarine nursery dependency can result in self-recruitment, where larvae remain in or return to their natal estuaries rather than dispersing widely via ocean currents (Pattrick and Strydom 2014, Tang et al. 2023). Moreover, coastal geomorphology, such as bays, estuaries and offshore islands, can create hydrodynamic barriers, such as eddies or retention zones, that disrupt larval dispersal (Pineda et al. 2007). In Central Vietnam, the intricate coastlines of provinces like Hue, Nghe An and Quang Binh result in significant implications for marine biodiversity and fisheries management (Tran and Le 2012). The coastline is highly irregular, characterised by numerous bays, river estuaries and lagoons, such as the Ca Estuary in Nghe An and the Gianh, Nhat Le Estuary in Quang Binh and the Tam Giang-Cau Hai Lagoon in Hue. This lagoon, with its narrow inlets and complex network of channels, creates a semi-enclosed environment with weak tidal currents and limited exchange with the open sea (Tran et al. 2010, Hoang and Tran 2020). The nearshore currents at the estuaries of the Ca River, Gianh River and Nhat Le River are strongly influenced by the monsoon regime, exhibiting an irregular and low-amplitude tidal regime (Tran and Nguyen 2014, Nguyen et al. 2021, Tran et al. 2024). These localised disruptions can lead to subtle genetic differentiation, even in species with high dispersal potential. Thus, a comprehensive understanding of both broad-scale connectivity and local genetic structuring is critical for predicting the responses of *A. latus* to ongoing environmental changes.

In this study, we investigated the genetic diversity, structure and demographic history of *A. latus* populations distributed across Central Vietnam, using mtCR sequences and a suite of population genetic analyses. Specifically, we aimed to: 1) quantify levels of genetic variation within and amongst regions; 2) determine whether population structuring exists along the Central Vietnamese coast; and 3) reconstruct the evolutionary history of *A. latus* populations to detect historical demographic events such as bottlenecks or expansions. The findings of this study not only fill a critical gap in our understanding of *A. latus* population genetics in Vietnam, but also provide essential data for the development of regionally informed conservation and fishery management policies.

## Materials and methods

### Sample collection total DNA extraction

Between January 2023 and June 2024, a total of 125 fish samples were collected from three regions in Central Vietnam: Nghe An, Quang Binh and Hue. To ensure a comprehensive representation, repeated sampling was performed at specific locations (Table 1). In total, 40 samples were gathered from Nghe An (NA1, NA2), 43 from Quang Binh (QB1, QB2) and 42 from Hue (HUE1, HUE2). These specimens were examined for their morphological traits and tissue samples — including muscle bone and fin — were taken for genetic analysis at the Laboratory of Zoology, Faculty of Biology, University of Education, Hue University, following ethical standards for animal research. The samples were obtained through direct collaboration with local fishermen who assisted in collecting specimens from active fishing operations in the study areas. This approach ensured the inclusion of diverse and representative samples from across the surveyed locations. The study received approval from the Animal Ethics Committee at Hue University (HUVN0036) and all experimental procedures adhered to the guidelines outlined in the Handbook for the Welfare of Laboratory Animals (Nguyen et al. 2022).

*A. latus* was identified from the fish samples using dichotomous keys outlined by Iwatsuki (2013), which were cross-referenced for species confirmation and selection. To ensure consistency with international standards, the classification system proposed by Fricke et al. (2023) was applied. The identification process involved a detailed comparison of key morphological traits, including body structure, fin counts, spines and specific meristic indices, with standard species descriptions to ensure accurate and reliable identification (Iwatsuki 2013).

Genomic DNA was extracted from 125 fish specimens identified as *A. latus* through morphological analysis (Table 1). Pectoral fin tissues were dissected, placed in 96% ethanol within Eppendorf tubes and stored at −20°C until further use. The extraction of total DNA was performed using the GenJET Genomic DNA Purification Kit (Thermo Fisher Scientific) following the manufacturer's protocol. The quality of the extracted DNA was assessed using 0.8% agarose gel electrophoresis with Safe-dye™ and the bands were visualised under UV light.

### DNA amplification and sequencing protocols

The PCR reaction mix (30 µl) included 3 µl of DNA template, 15 µl of 2× PCR Master Mix, 3 µl each of the forward (DL-S, 5’-CCCACCACTAACTCCCAAAGC-3’) and reverse (DL-R, 5’-TTAACTTATGCAAGCGTCGAT-3’) primers (Gao et al. 2019) and 9 µl of distilled water. The amplification was carried out in an ESCO thermal cycler with the following cycling protocol: an initial denaturation at 94°C for 5 minutes, followed by 30 cycles consisting of 94°C for 1 minute, 52°C for 40 seconds and 72°C for 40 seconds and a final extension step at 72°C for 5 minutes. The resulting PCR products were evaluated by agarose gel electrophoresis (0.8%–1.4%) with Safe-dye™ (1 µl/20 ml gel) and bands were visualised using a UV transilluminator. DNA sequencing was conducted using the Sanger method on an ABI Prism DNA Analyzer (Applied Biosystems, USA) at 1st Base (Malaysia).

### Phylogenetic evaluation

To verify the identity and similarity of the mtCR gene segment sequences, the Basic Local Alignment Search Tool (BLAST) was used. Once validated, the sequences were edited and aligned using MEGA X software (Kumar et al. 2018). We also included the *mtCR* sequences from NCBI GenBank in our analysis. A detailed list of *A. latus* and their corresponding accession numbers can be found in Suppl. material 1.

The alignments were then refined using Bioedit (Hall 1999). These alignments were saved in various file formats such as FASTA, MEGA, ARLQUIN and NEXUS for ease of subsequent analyses. Haplotype analysis was conducted using DnaSP 6 (Rozas et al. 2017). A Bayesian phylogenetic tree was constructed in BEAST 2.7 (Bouckaert et al. 2019), applying the HKY substitution model and the coalescent Bayesian skyline tree prior. The Markov Chain Monte Carlo analysis ran for 10,000,000 generations, with a 10% burn-in. The resulting phylogenetic trees were visualised and adjusted using the iTOL web tool (https://itol.embl.de/, Letunic and Bork (2024)). The Median Haplotype Network was subsequently constructed with the help of POPART 1.7 (Leigh and Bryant 2015).

### Genetic diversity and population structure pattern

The genetic parameters, including the number of variable sites (S), mutations (η), average nucleotide differences (k), haplotype count (h), nucleotide diversity (π) and haplotype diversity (Hd), were determined within and between each sampling region with DnaSP 6 (Rozas et al. 2017). Population expansion patterns of *A. latus* were examined through pairwise mismatch distribution and neutrality tests by calculating Fu’s Fs and Tajima’s D using DnaSP and Arlequin 3.5 software (Excoffier and Lischer 2010). Fu’s Fs evaluates the distribution of haplotypes, while Tajima’s D assesses pairwise sequence differences to infer changes in allele frequencies. The genetic divergence (fixation index, Fst) and variability within and between populations were analysed through analysis of molecular variance (AMOVA) using Arlequin 3.5 (Excoffier and Lischer 2010).

The population dynamics of *A. latus* were analysed by creating a Bayesian Skyline Plot (BSP) using BEAST 2.7, with a stepwise constant function. Substitution models for mtCR partition was estimated using jModelTest2 (Darriba et al. 2012). We selected the HKY + G nucleotide substitution model and conducted MCMC simulations for 100 million generations, sampling every 1000 generations. The first 10% of the samples were discarded as burn-in. Since no specific mutation rate for the *A. latus mtCR* was available, we calibrated the analysis using the *A. pacificus mtCR* mutation rate of 3.6 × 10^−8^ per site per year (Islam et al. 2022). The resulting BSP was visualised in TRACER v.1.7.2, focusing on a period beginning around 50,000 years ago.

## Results

### Phylogenetic relationships of A. latus based on the mtCR

**Figure 1.**
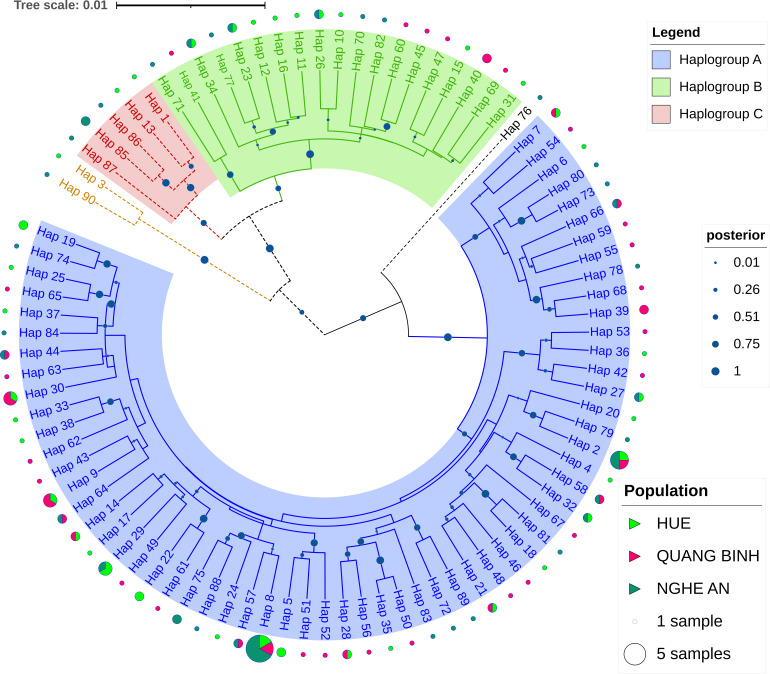
The Bayesian phylogenetic tree illustrating the relationship between *A. latus* from Central Vietnam. Posterior probabilities were used to assess the robustness of the tree. *A. latus* from Central Vietnam is labelled with numerical symbols at their ends, based on the haplotype order. The accession numbers on NCBI GenBank for the *A. latus* included in the phylogenetic analysis are provided in Suppl. material 1. Haplogroup A is denoted in light blue, haplogroup B in light green and haplogroup C in light red. Other haplogroups are included to provide a comprehensive view of all mitochondrial haplogroups. The scale bar in the tree represents the average number of substitutions per site. The posterior probability for each branch is indicated by dots, with sizes corresponding to the probability values.

**Figure 2.**
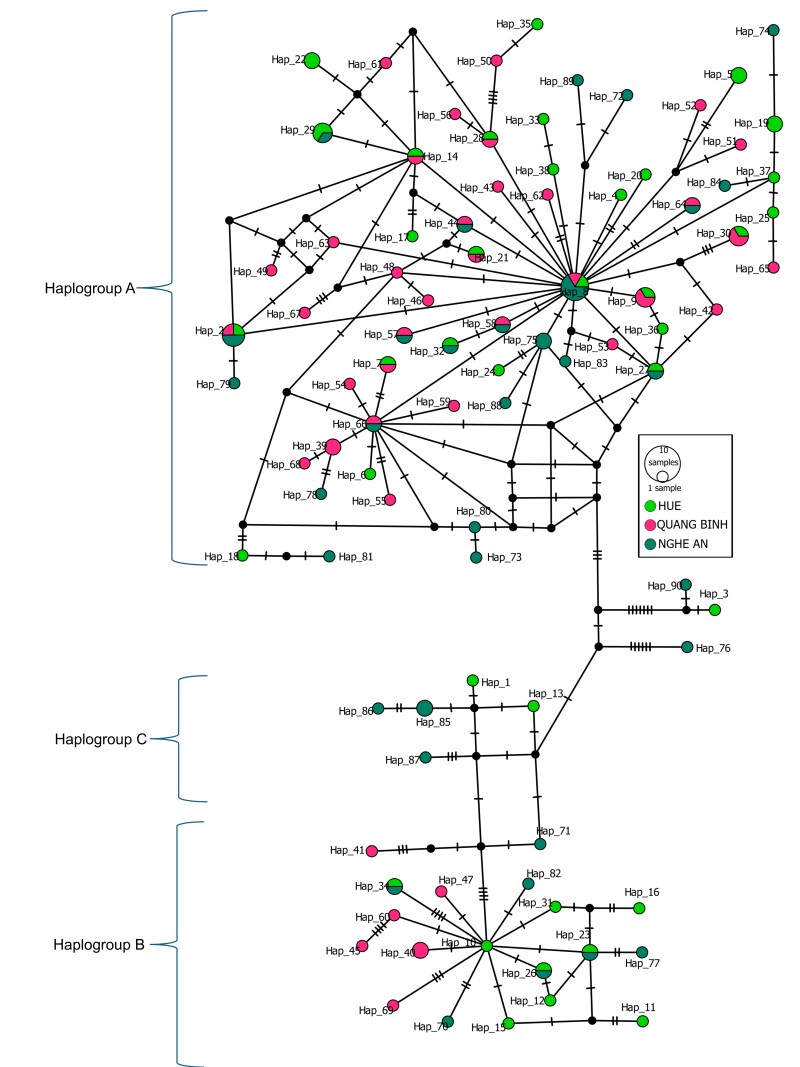
The median-joining haplotype network of *A. latus* from Central Vietnam illustrating the relationships amongst mtCR haplotypes. Each circle denotes a unique haplotype and the size of the circle is proportional to the sample total frequency. Each branch connecting different haplotypes illustrates a number of nucleotide changes by the number of black dashes on the branches. Colours represent sample geographic origins as indicated by the legend.

From 125 sequences, 90 haplotypes were identified, with no dominant haplotype observed. Of these, 71 haplotypes were unique, while 19 were shared: 17 haplotypes were shared between two populations and two haplotypes were shared amongst three populations. A Bayesian phylogenetic tree analysis, based on the mtCR haplotypes of *A. latus* (Fig. 1), revealed three main clades. The first clade, belonging to haplogroup A, encompassed the majority of individuals from the sampled population. The second clade comprised 19 haplotypes assigned to haplogroup B. The third clade, corresponding to haplogroup C, formed a distinct group closely related to haplogroup B, consisting of five *A. latus* individuals. Notably, certain *A. latus* haplotypes, such as 76, 3 and 90, exhibited monophyletic divergence and did not cluster closely with any of the aforementioned haplogroups, indicating unique genetic characteristics and these remained unclassified. To further explore the genetic diversity within the identified haplogroups, a median-joining haplogroup network was constructed using the mtCR haplotypes (Fig. 2). Most haplotypes were classified under haplogroup A, while 19 belonged to haplogroup B and five were assigned to haplogroup C. The haplotypes did not segregate according to sampling locations; however, the majority were grouped in haplogroup A, forming a large central network with the high-frequency haplotype Hap_8 (six samples). In contrast, the central haplotype of haplogroup B, Hap_10 (one sample), exhibited a lower frequency. Overall, the results of the Bayesian phylogenetic tree and the haplogroup network analyses were highly consistent with each other.

### Genetic diversity of A. latus in Central Vietnam

In this study, we examined genetic diversity across three regions: Hue, Quang Binh and Nghe An, based on various genetic parameters (Table 2). A total of 125 samples were analysed, with 42 from Hue, 43 from Quang Binh and 40 from Nghe An. The number of variable sites (S) ranged from 55 to 63 across the regions and the total number of mutations (η) varied between 57 and 64. The overall nucleotide diversity (π) across all samples was 0.01910 ± 0.00127, with the highest value observed in Hue (0.02121 ± 0.00208) and the lowest in Quang Binh (0.01669 ± 0.00235). The average number of nucleotide differences (k) was highest in Hue (9.9698), with the other two regions showing lower values. In terms of haplotype diversity, a total of 90 haplotypes were identified across all samples, with the highest number found in Quang Binh (39), followed by Hue (38) and Nghe An (34). The haplotype diversity (Hd) was similarly high across all regions, with values ranging from 0.988 to 0.996 and the overall diversity was 0.993 ± 0.002. These findings suggest that there is a relatively high degree of genetic variation within the populations, with some regional differences in diversity.

The results from the AMOVA analysis of *A. latus* populations in Central Vietnam (Table 3) revealed that the genetic variation between populations was minimal. The amongst-population variance component (Va) was found to be 0.00014, accounting for just 0.03 of the total genetic variation. In contrast, the within-population variance component (Vb) was significantly higher at 0.49661, which accounted for 99.97% of the total genetic variation. These results indicate that the majority of genetic variation in *A. latus* is found within populations, rather than between populations. The fixation index (Fst) value of 0.00029 (p = 0.39188) suggests weak genetic differentiation amongst the populations studied.

Further analysis of pairwise Fst values revealed low, but significant genetic differentiation between the populations (Table 4). Specifically, the pairwise Fst values were highest between Quang Binh and Nghe An (Fst = 0.00159), followed by Hue and Nghe An (Fst = 0.00035). However, the differentiation between Hue and Quang Binh was considerably lower (Fst = -0.00101). The P-values for all pairwise comparisons were non-significant, indicating that, while genetic differentiation exists, it is not statistically significant at the population level. These results suggest that, although there is some degree of genetic variation between populations, the populations of *A. latus* in Central Vietnam are relatively interconnected with limited barriers to gene flow.

### Evolutionary and historical patterns of A. latus

To investigate the evolutionary dynamics and historical patterns of *A. latus* in Central Vietnam, we conducted a comprehensive genetic analysis using neutrality tests, mismatch distribution and Bayesian Skyline Plot approaches across three regions: Hue, Quang Binh and Nghe An. Tajima's D values were negative for all regions, suggesting a population expansion or selective sweep (Tajima 1989). Furthermore, all regions exhibited highly significant negative Fu’s Fs values (p < 0.00001) (Table 2), reinforcing the evidence of population expansion across the studied areas (Fu 1997).

**Figure 3.**
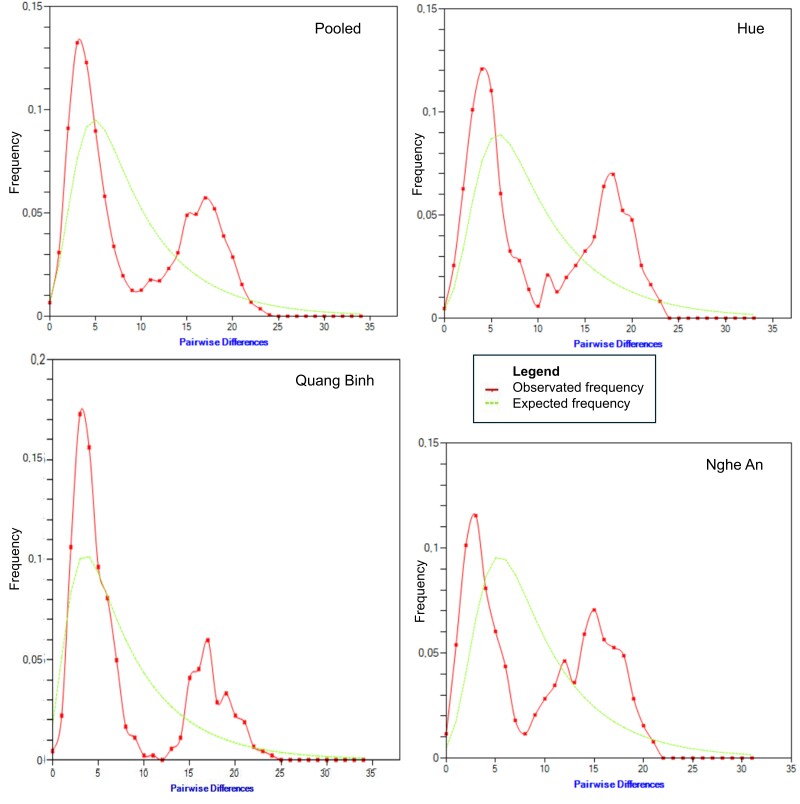
Mismatch distribution plot, based on *A. latus* mtCR data, pooled data of Central Vietnam and individual sites at Hue, Quang Binh and Nghe An.

The mismatch distribution plots, based on mtCR data for the *A. latus* populations from Central Vietnam (Fig. 3), provide valuable information for their demographic history. The pooled data and each regional plot display a multimodal distribution, with the observed frequency (red) showing multiple peaks, particularly between 5-15 pairwise differences, alongside a secondary peak around 15-20 differences. This pattern contrasts with the expected frequency under a sudden expansion model (green), an unimodal curve peaking at higher differences (around 5-10). The divergence between observed and expected distributions, especially the extended tail towards 35 differences, suggests a complex population history that may involve stable population sizes over time or the presence of distinct subpopulations with limited gene flow (Harpending 1994). The similarity across the three regions indicates a shared demographic pattern, though variations in peak heights and tail lengths hint at localised differences in genetic structure or historical events (Schneider and Excoffier 1999).

**Figure 4.**
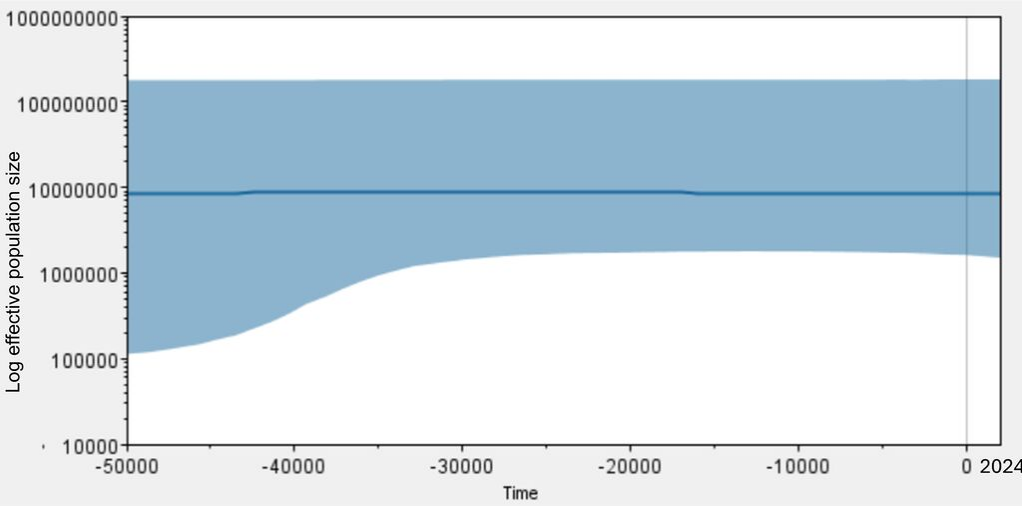
The Bayesian Skyline Plot (BSP) depicts the population dynamics of *A. latus* over time, spanning from 50,000 BC to the present.

In addition, the Bayesian Skyline Plot analysis, based on the median effective population size (Ne) (Fig. 4), reveals a stable demographic history for the *A. latus* population over the past 50,000 years. The population experienced a slight increase by 40,000 BC, indicating a period of little expansion. From 40,000 to 15,000 BC, Ne remained relatively constant, suggesting a long phase of population stability. However, a slight decline is observed from 15,000 BC to the present. To sum up, these findings reveal a complex interplay of historical expansion, prolonged stability and recent decline in *A. latus* populations.

## Discussion

The genetic structure and population dynamics of *A. latus* in Central Vietnam, as explored through mtDNA analysis, reveal a picture of genetic diversity and significant insights into the evolutionary history of this ecologically and economically important species. Through the mtCR markers, our study provides a comprehensive understanding of the expansion, origin and connectivity of *A. latus* populations along the central Vietnamese coast. The findings enhance our knowledge of the *A. latus* population genetics and offer critical insights for future conservation and fisheries management strategies. Specifically, our analysis of mtCR markers revealed significant patterns in genetic diversity and population connectivity.

Our study found that *A. latus* populations in Central Vietnam exhibit considerable genetic diversity, as reflected in high haplotype diversity across the three sampled regions: Hue, Quang Binh and Nghe An. These results are consistent with the findings of previous studies on marine species, which suggest that high levels of genetic diversity are crucial for the adaptation of populations to changing environmental conditions (Frankham et al. 2010, Liu et al. 2021). The genetic diversity observed in this study suggests that *A. latus* populations have been able to maintain their genetic integrity despite potential ecological and anthropogenic pressures, including habitat degradation and overfishing. For instance, such high genetic variability is particularly important in marine species, where adaptation to fluctuating coastal environments, such as variations in temperature, salinity and food availability, can determine the resilience of populations (Wang et al. 2024, Yang et al. 2024).

Genetic analysis of *A. latus* populations in Central Vietnam revealed little genetic differentiation amongst the three sampled regions, with pairwise Fst values ranging from -0.00101 - 0.00035 (p > 0.05), indicating low population structuring (Frankham et al. 2010). The absence of notable genetic variation amongst populations of *A. latus* observed in our research may be attributed to substantial gene flow occurring during their pelagic phase. This observation aligns with the life history of *A. latus*, characterised by an extended pelagic larval and early juvenile period, facilitating population mixing (Chang et al. 2002). Evidence of gene flow across these regions suggests that populations are not entirely isolated, likely due to the dispersal of planktonic larvae facilitated by coastal currents and the north-south ocean current in the East Sea (Doan 2022). Oceanographic factors, particularly the north-south current with velocities of 1.05–1.26 m/s along the Central Vietnam coast, play a vital role in larval transport across vast distances, maintaining population connectivity (Wang et al. 2024). Additionally, seasonal coastal currents influenced by monsoon winds enhance larval dispersal to estuarine nursery grounds, critical for population recruitment. The surface current during winter, predominantly flowing from north to south along the Vietnam coast (MRCC, Vietnam 2025), coincides with the spawning and dispersal season of yellowfin seabream larvae and juveniles (Tran et al. 2019). All over, this structure is similar to the results of the study of *A. latus* fish populations in western Japan by Syazni et al. (2015) and around Taiwan by Jean et al. (2000). Overall, these observations suggest a high degree of gene flow amongst *A. latus* populations in Central Vietnam.

Additionally, populations in Central Vietnam have very low Fst values when compared to southern China populations (Hainan, Guangdong, Fujian), ranging from -0.00015 to 0.00649 with p-values above 0.05 (Table 5), indicating no significant genetic differences. This suggests strong gene flow, likely due to environmental factors like ocean currents. Prior research indicates that *A. latus* spawns from September to January in the South China Sea (Wang et al. 2020). This spawning period coincides with the winter monsoon season, a critical factor for larval dispersal. This pattern of connectivity is consistent with other studies on marine species that highlight the role of ocean currents in shaping the population structure of marine organisms. For example, genetic studies on *A. pacificus* populations across the West Pacific suggest that ocean currents, such as the Kuroshio Current, significantly influence larval dispersal and connectivity between populations (Islam et al. 2022). The China Coastal Current has been identified as a key factor supporting larval dispersal, maintaining gene flow (Li et al. 2025). In contrast, when comparing the *A. latus* population in western Japan with that in Central Vietnam, the Fst index is at a moderate and significant level of 0.01783 (p < 0.05) (Table 5), reflecting that the connectivity between these two regions is somewhat weak. This weaker connectivity could be explained by the very large geographical distance between the two populations, which likely limits gene flow. Unlike the proximity and shared ocean currents facilitating connectivity with southern China, the distance to Japan introduces a barrier, reducing genetic exchange.

However, despite this connectivity driven by ocean currents, local environmental factors may also influence population differentiation. The genetic structure of *A. latus* also suggests that local environmental factors, including the availability of suitable estuarine and coastal habitats, may contribute to population differentiation at smaller scales. Studies of other marine species have shown that self-recruitment, where larvae are retained in their natal habitats, can also contribute to genetic differentiation between populations despite the potential for long-distance larval dispersal (Jones et al. 1999). In the case of *A. latus*, a shallow-water species living around rocky reefs, coral reefs, estuaries and coastal lagoons (Song et al. 2021, Tang et al. 2023), both local retention and long-distance dispersal mechanisms are at play. This dual mechanism may lead to some degree of population isolation, driven by habitat-specific conditions, such as variations in salinity and water temperature across the different coastal regions of Vietnam.

The demographic patterns indicated by the neutrality tests, including significant negative values of Fu’s Fs and Tajima’s D, along with mismatch distribution and Bayesian Skyline Plot analyses, suggest a slight historical population expansion. As a result, this expansion pattern, as observed in many marine taxa following bottleneck events or climatic shifts (Song et al. 2021), was likely driven by favourable climatic conditions at the end of the Pleistocene, when global sea levels rose, leading to the overflow of coastal areas and the creation of new habitats for marine species (Ludt and Rocha 2014, Boissin et al. 2016). These environmental changes facilitated the recovery and growth of marine populations across the Indo-Pacific (Sun et al. 2012, Ludt and Rocha 2014, Delrieu-Trottin et al. 2017, Islam et al. 2022, Wang et al. 2022, Oosting et al. 2023, Li et al. 2025). As a shallow-water species inhabiting coral reefs, estuaries and coastal lagoons, *A. latus* likely benefitted from this recovery, which was supported by the persistence of suitable breeding and feeding grounds in coastal and estuarine habitats, critical for maintaining population stability (Francisco et al. 2009, Sourinejad et al. 2015, Song et al. 2021, Wang et al. 2024).

The high genetic diversity and evidence of historical population expansion in *A. latus* in Central Vietnam suggested moderate adaptive potential to environmental changes (Song et al. 2021, Thomson et al. 2021). However, this resilience is threatened by ongoing habitat degradation, overfishing (Matsuishi 2025) and climate change impacts, which may reduce genetic diversity and population stability (Craig 2012, Von der Heyden et al. 2014). For instance, the loss of critical habitats like coral reefs, mangroves and seagrass beds underscores the need for urgent conservation measures. Specifically, Nguyen et al. (2019) reported a low resilience index (0.369) for coral reefs on Hon La Island, driven by high oil concentrations and turbidity, underscoring the need for integrated coastal management and marine protected areas (Nguyen et al. 2019). Likewise, Le (2020) found an average coral cover of only 19.8% across six sites in Central Vietnam, with widespread bleaching and disease, demanding detailed studies to guide effective protection strategies for coastal coral reef ecosystems (Le 2021). Moreover, the degradation of marine ecosystems, including the loss of 70% of mangrove forests and 40–60% of seagrass beds across Vietnam, requires robust policy enforcement and community-driven restoration initiatives to ensure long-term ecological sustainability (Pham and Khanal 2024). These findings underscore the critical need to protect key habitats, such as coral reefs, mangroves, seagrass beds and estuarine areas, which serve as essential breeding and nursery grounds for *A. latus* (Nagelkerken et al. 2000, Hejnowicz et al. 2015, Nordlund et al. 2018). Furthermore, the influence of ocean currents on population connectivity in this study, as evidenced by the little genetic difference pattern, highlights the need for regionally coordinated fisheries management to account for transboundary population linkages (Sills et al. 2018, Roberts et al. 2024). Therefore, integrating genetic data into management strategies, such as strengthening management of marine protected areas, based on genetic diversity hotspots (e.g. Hai Van - Son Tra, Con Co, Hon La - Hon Co, Hon Ngu - Dao Mat), will enhance the sustainability of *A. latus* populations in Central Vietnam and the broader Western Pacific Region.

In conclusion, effective management strategies for *A. latus* must account for its complex population structure and connectivity across the Western Pacific to safeguard its ecological and economic importance in the region.

## Conclusions

To sum up, high genetic diversity and weak population differentiation in *A. latus* across Central Vietnam are driven by ocean currents and local retention. Historical expansion, stability and recent decline highlight the need for habitat protection and regionally coordinated fisheries management to ensure sustainability.

## Supplementary Material

7335A04D-2EA2-5884-BC3E-FCF057D48787 10.3897/BDJ.13.e164274.suppl1 Supplementary material 1 Supplementary Table S1 Data type GenBank accession numbers Brief description List of *A. latus* and their GenBank accession numbers used in the phylogenetic analysis. File: oo_1360634.pdf https://binary.pensoft.net/file/1360634 Nguyen Ty, Bui Van Loi, Hoang Le Thuy Lan, Tran Thi Kim Anh, Nguyen Xuan Huy, Tran Van Giang

## Figures and Tables

**Table 1.** The fish samples were used for total DNA extraction.

**No.**	**Location**	**Sampling site**	**Latitution**	**Number of Samples**	**Sample Code**
1	Hue	HUE1	16°35'48.19"N, 107°37'13.86"E	22	1001, 1002, 1003, 1004, 1005, 1006, 1136, 1137, 1138, 1139, 1140, 1141, 1142, 1143, 1144, 1148, 1149, 1150, 1151, 1152, 1153, 1189
		HUE2	16°21'59.57"N, 107°56'30.14"E	20	2121, 2122, 2124, 2125, 2126, 2127, 2128, 2129, 2511, 2512, 2513, 2514, 2515 2516, 2601, 2602, 2603, 2604, 2605, 2606
2	Quang Binh	QB1	17°42'46.81"N, 106°30'26.39"E	21	4101, 4102, 4103, 4104, 4105, 4107, 4108, 4109, 4110, 4111, 4157, 4158, 4159, 4160, 4161, 4162, 4163, 4164, 4165, 4166, 4167
		QB2	17°29'10.43"N, 106°38'9.87"E	22	3122, 3123, 3124, 3125, 3126, 3127, 3128, 3129, 3130, 3131, 3132, 3133, 3134, 3135, 3136, 3137, 3138, 3139, 3140, 3141, 3142, 3143
3	Nghe An	NA1	18°58'19.57"N, 105°38'5.53"E	20	5213, 5214, 5215, 5216, 5217, 5218, 5219, 5220, 5221, 5222, 5223, 5224, 5225, 5226, 5227, 5228, 5229, 5230, 5231, 5232
		NA2	18°48'49.86"N, 105°45'7.77"E	20	6201, 6202, 6203, 6204, 6205, 6206, 6207, 6208, 6209, 6401, 6402, 6403, 6404, 6405, 6406, 6407, 6408, 6410, 6411, 6412

**Table 2.** Genetic diversity of *A. latus* populations in Central Vietnam, based on *mtCR* sequences data.

**Value**	**Hue**	**Quang Binh**	**Nghe An**	**Total**
Number of samples	42	43	40	125
Number of variable sites (*S*)	63	62	55	95
Number of mutations (*η*)	64	63	57	99
Average number of nucleotide differences (*k*)	9.9698	7.8461	9.1359	8.9764
Nucleotide diversity per site (*π*)*	0.02121 ± 0.00208	0.01669 ± 0.00235	0.01944 ± 0.00206	0.01910 ± 0.00127
Number of haplotypes (*h*)	38	39	34	90
Haplotype (gene) diversity (*Hd*)*	0.995 ± 0.006	0.996 ± 0.006	0.988 ± 0.010	0.993 ± 0.002
Tajima’s *D* **	-1.14126 (0.115)	-1.61193 (0.030)	-1.05042 (0.143)	-1.26787 (0.096)
Fu’s *F*_s_	-24.61588***	-24.90301***	-21.90654***	-23.80848***

Note: *, presented as the mean ± standard deviation; **, presented as the value (p-value); ***, p < 0.00001.

**Table 3.** AMOVA of *A. latus* populations in Central Vietnam.

**Source**	**Degree of** **freedom**	**Sum of** **squares**	**Variance** **components**	**Percentage of** **total variance (%)**	**Fixation index (*F*_st_)**
Amongst populations	2	1.005	0.00014 Va	0.03	0.00029 (P = 0.39188)
Within populations	122	60.587	0.49661 Vb	99.97	
Total	124	61.592	0.49676		

**Table 4.** The *F*_st_ of *A. latus* populations in Central Vietnam.

**Population**	**Hue**	**Quang Binh**	**Nghe An**
Hue	***	0.66726	0.38353
Quang Binh	-0.00101	***	0.24621
Nghe An	0.00035	0.00159	***

Note: The *F*_st_ is shown below the asterisk (***) symbol while the *P*-value is shown above it.

**Table 5.** The Fst values between *A. latus* from Central Vietnam and several *A. latus* from around the world. The accession numbers on NCBI GenBank for the *A. latus* included in the genetic difference analysis are provided in Suppl. material 1. Symbol * denotes statistical significance (P < 0.05).

Population	Hainan	Guangdong	Fujian	Western Japan
Hue	0.00326	0.00321	-0.00015	0.00911 *
Quang Binh	0.00311	0.00306	0.00311	0.02567 *
Nghe An	0.00649	0.00638	0.00649	0.01926 *
Central Vietnam	0.00463	0.00456	0.00349	0.01783 *
