# Supplementary material for: Uncovering the genetic structure and evolutionary history of Acanthopagrus latus (Actinopterygii, Sparidae) using mitochondrial DNA and multivariate analyses: A case study of coastal populations in Central Vietnam
Source: Biodivers Data J. 2025 Aug 22;13:e164274. doi: 10.3897/BDJ.13.e164274 (PMC12397771; doi:10.3897/BDJ.13.e164274)
Supplement: Supplementary material 1 — Supplementary Table S1 [file bdj-13-e164274-s001.pdf]

Supplementary Table S1. List of *A. latus* and their GenBank accession numbers used in the phylogenetic analysis

No.	Haplotypes	Sample No.	Acccession number	Reference
1	Hap_1	1001	PV693208	This study
2	Hap_2	1002; 4107; 5215; 6204	PV693209; PV693255; PV693295; PV693316	This study
3	Hap_3	1003	PV693210	This study
4	Hap_4	1004	PV693211	This study
5	Hap_5	1005; 1006	PV693212; PV693213	This study
6	Hap_6	1136	PV693214	This study
7	Hap_7	1137; 4161	PV693215; PV693264	This study
8	Hap_8	1138; 3135; 5220; 6201; 6404; 6406	PV693216; PV693284; PV693300; PV693313; PV693325; PV693327	This study
9	Hap_9	1139; 3133; 3141	PV693217; PV693282; PV693290	This study
10	Hap_10	1140	PV693218	This study
11	Hap_11	1141	PV693219	This study
12	Hap_12	1142	PV693220	This study
13	Hap_13	1143	PV693221	This study
14	Hap_14	1144; 3140	PV693222; PV693289	This study
15	Hap_15	1148	PV693223	This study
16	Hap_16	1149	PV693224	This study
17	Hap_17	1151	PV693225	This study
18	Hap_18	1150	PV693226	This study
19	Hap_19	1152; 2511	PV693227; PV693238	This study
20	Hap_20	1153	PV693228	This study
21	Hap_21	1189; 3137	PV693229; PV693286	This study
22	Hap_22	2121; 2127	PV693230; PV693235	This study
23	Hap_23	2122; 6412	PV693231; PV693332	This study
24	Hap_24	2124	PV693232	This study
25	Hap_25	2125	PV693233	This study
26	Hap_26	2126; 5223	PV693234; PV693303	This study
27	Hap_27	2128; 5216	PV693236; PV693296	This study
28	Hap_28	2129; 3143	PV693237; PV693292	This study
29	Hap_29	2512; 2514; 6207	PV693239; PV693241; PV693319	This study
30	Hap_30	2513; 4102; 3132	PV693240; PV693251; PV693281	This study
31	Hap_31	2515	PV693242	This study
32	Hap_32	2516; 6403	PV693243; PV693324	This study
33	Hap_33	2601	PV693244	This study
34	Hap_34	2602; 5218	PV693245; PV693298	This study
35	Hap_35	2603	PV693246	This study
36	Hap_36	2604	PV693247	This study
37	Hap_37	2605	PV693248	This study
38	Hap_38	2606	PV693249	This study
39	Hap_39	4101; 4109	PV693250; PV693257	This study
40	Hap_40	4103; 3125	PV693252; PV693274	This study
41	Hap_41	4104	PV693253	This study
42	Hap_42	4105	PV693254	This study

43	Hap_43	4108	PV693256	This study
44	Hap_44	4110; 6401	PV693258; PV693322	This study
45	Hap_45	4111	PV693259	This study
46	Hap_46	4157	PV693260	This study
47	Hap_47	4158	PV693261	This study
48	Hap_48	4159	PV693262	This study
49	Hap_49	4160	PV693263	This study
50	Hap_50	4162	PV693265	This study
51	Hap_51	4163	PV693266	This study
52	Hap_52	4164	PV693267	This study
53	Hap_53	4165	PV693268	This study
54	Hap_54	4166	PV693269	This study
55	Hap_55	4167	PV693270	This study
56	Hap_56	3122	PV693271	This study
57	Hap_57	3123; 6203	PV693272; PV693315	This study
58	Hap_58	3124; 5222	PV693273; PV693302	This study
59	Hap_59	3126	PV693275	This study
60	Hap_60	3127	PV693276	This study
61	Hap_61	3128	PV693277	This study
62	Hap_62	3129	PV693278	This study
63	Hap_63	3130	PV693279	This study
64	Hap_64	3131; 6208	PV693280; PV693320	This study
65	Hap_65	3134	PV693283	This study
66	Hap_66	3136; 6411	PV693285; PV693331	This study
67	Hap_67	3138	PV693287	This study
68	Hap_68	3139	PV693288	This study
69	Hap_69	3142	PV693291	This study
70	Hap_70	5213	PV693293	This study
71	Hap_71	5214	PV693294	This study
72	Hap_72	5217	PV693297	This study
73	Hap_73	5219	PV693299	This study
74	Hap_74	5221	PV693301	This study
75	Hap_75	5224; 6410	PV693304; PV693330	This study
76	Hap_76	5225	PV693305	This study
77	Hap_77	5226	PV693306	This study
78	Hap_78	5227	PV693307	This study
79	Hap_79	5228	PV693308	This study
80	Hap_80	5229	PV693309	This study
81	Hap_81	5230	PV693310	This study
82	Hap_82	5231	PV693311	This study
83	Hap_83	5232	PV693312	This study
84	Hap_84	6202	PV693314	This study
85	Hap_85	6205; 6206	PV693317; PV693318	This study
86	Hap_86	6209	PV693321	This study
87	Hap_87	6402	PV693323	This study
88	Hap_88	6405	PV693326	This study
89	Hap_89	6407	PV693328	This study
90	Hap_90	6408	PV693329	This study

	AF549503 - AF549524	Liu H., S. Jiang, 2002
	AB916774 - AB916800	Syazni K.A., S. Tomano, et al., 2015
